# Acromegaly presenting with myelopathy due to ossification of posterior longitudinal ligament: a case report

**DOI:** 10.1186/s12891-021-04232-6

**Published:** 2021-04-14

**Authors:** Daisuke Kamakura, Katsunori Fukutake, Kazumasa Nakamura, Shintaro Tsuge, Keiji Hasegawa, Naobumi Tochigi, Akihito Wada, Tetsuo Mikami, Hiroshi Takahashi

**Affiliations:** 1 Department of Orthopedic Surgery, Toho University Omori Medical Center, 6-11-1, Omori-Nishi, Ota-Ku, Tokyo, 143-8541 Japan; 2 Department of Surgical Pathology, Toho University Omori Medical Center, 6-11-1, Omori-Nishi, Ota-Ku, Tokyo, 143-8541 Japan

**Keywords:** Acromegaly, Ossification of posterior longitudinal ligament, Case report

## Abstract

**Background:**

Acromegaly is a rare disease caused by high serum levels of growth hormone (GH) and insulin-like growth factor 1 (IGF-1), often originating from a pituitary adenoma. Spinal and peripheral joint abnormalities are caused by these hormonal hypersecretions. In particular, the response to GH is involved in the onset of ossification of the spinal ligament in vitro, especially ossification of the posterior longitudinal ligament (OPLL). However, because acromegaly and OPLL are rare diseases, we seldom encounter them in combination. To the best of our knowledge in the English-language literature, this is the first reported case of acromegaly presenting with thoracic myelopathy due to OPLL.

**Case presentation:**

A 47-year-old woman presented with lower extremity weakness and paresthesia, gait disorder, and bladder disorder without any trauma. The patient’s most remarkable symptom was paraplegia, and we diagnosed myelopathy due to cervical and thoracic OPLL. Furthermore, we suspected acromegaly because of the characteristic facial features, and we found a pituitary adenoma by contrast-enhanced MRI. Cervical and thoracic decompression, posterior fixation, and pituitary adenoma resection were performed.

**Conclusion:**

We report a case of acromegaly that was detected after the diagnosis of OPLL. The main challenge in acromegaly is delayed in diagnosis. Even in this case, the facial features characteristic of acromegaly had appeared at least 9 years ago. Early diagnosis and treatment of acromegaly improve prognosis and reduce exposure to GH and IGF-1 through early intervention and seem to suppress the progression of ligament ossification. Orthopedic surgeons and neurosurgeons need to keep in mind that acromegaly is associated with bone/joint lesions and ossification of the spinal ligament and should aim to diagnose acromegaly early.

## Background

Acromegaly is a rare disease caused by high serum levels of growth hormone (GH) and insulin-like growth factor 1 (IGF-1) usually from a pituitary adenoma. Spinal and peripheral joint abnormalities are caused by these hormonal hypersecretions. Furthermore, the posterior longitudinal ligament (OPLL) was first reported by Key in 1838 [1] and described in detail by Tsukimoto from our university in 1960 [2]. Breidahl reported 3 autopsy cases of this disease in Japanese patients in 1969 [3], and in Japan, we actively conducted research about OPLL as a disease unique to Japan. However, recent reports demonstrate that there is a certain morbidity rate in patients of other races. The overall prevalence of OPLL is 1.9–4.3% in the Japanese population, 0.8–3.0% in other Southeast Asian populations and 0.1–1.7% in North American and European populations, suggesting a sporadic distribution [4, 5]. The mechanism of ethnic background underlying this interracial difference is still unknown.

The association between acromegaly and OPLL has been previously pointed out in vitro. Ikegawa et al. reported that GH receptors are increased in patients with OPLL. Goto et al. reported that IGF-1 has a stronger effect on inducing ossification differentiation on OPLL patient ligament cells than on those of non-OPLL patients. These results suggest that excessive exposure to GH and IGF-1 is associated with the progression of ligament ossification [6, 7]. However, the annual incidence of acromegaly is very low [8, 9], and we seldom encounter these diseases. To the best of our knowledge, a case of myelopathy due to OPLL inapatient with acromegaly has not been reported previously in the English-language literature. We present the first reported case of acromegaly with myelopathy due to OPLL with some consideration of the relationship between acromegaly and OPLL and the early detection of acromegaly.

## Case presentation

### History

A 47-year-old woman presented with lower extremity weakness and paresthesia, gait disorder, and bladder and rectal disorder. Four months after the onset of numbness and motor weakness of the lower extremities, she presented to our hospital without any trauma. One week before presentation, the gait disorder and bladder and rectal disorder became apparent. The patient’s history included hypertension. There was no family history of acromegaly or OPLL.

### Physical examination

**Fig. 1:**
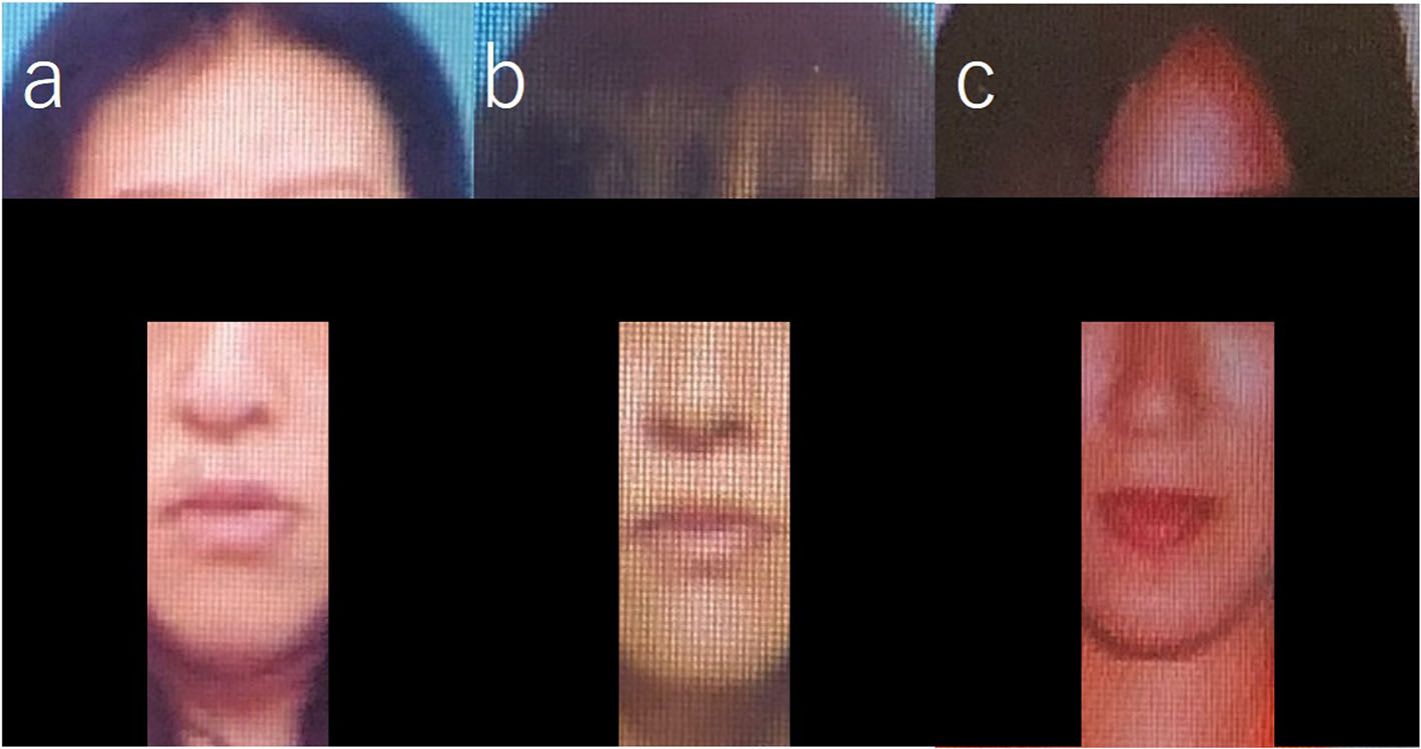
**a**-**c**: Facial appearance. **a**. At presenting our hospital. It showed acromegaly-like facial appearance, with a frontal protrusion, nose and lip enlargement, and mandibular protrusion. **b**. At 9 years ago from presenting our hospital. The features of acromegaly have already appeared. **c**. At 24 years ago from presenting our hospital. Almost normal facial appearance

She weighed 72 kg, her height was 162.0 cm, and her BMI was 27.4 kg/m2. Blood pressure at admission was 160/90 mmHg. Her facial appearance was acromegaly-like, with a frontal protrusion, nose and lip enlargement, and mandibular protrusion (Fig. 1a). We also observed thickening of the soft tissue of the palm and fingers and thickening of the Achilles tendon. There was no history of headache, sweating, or fatigue, and we did not observe Raynaud’s phenomenon or carpal tunnel syndrome.

In the upper extremities, no abnormal neurological findings were observed. However, weakness in both lower extremities (iliopsoas muscle MMT4, quadriceps femoris MMT4) was observed. Both lower extremity tendon reflexes were enhanced, and clonus was observed. Hypo-sensation (5/10) was observed below the umbilicus. These neurological findings suggested spinal cord lesions below the T10 level. She was barely able to walk on flat ground using two canes, and the Japanese Orthopedic Association score was 9 points (4–1–2-0-0-2).

### Blood tests

The complete blood count was normal. An evaluation of glycemic status revealed a fasting plasma glucose level of 115 mg/dL. The glycosylated hemoglobin level was 5.9%, and the plasma glucose level during the OGTT (75 g glucose) at 2 h was 153 mg/dL. These results ruled out type 2 diabetes. Hepatic function tests and renal function tests revealed no abnormalities except for high alkaline phosphatase (ALP) (432 IU/L). Estimations of serum electrolytes, including sodium, potassium, calcium, and phosphorous were within normal limits. Hormonal evaluation showed raised serum IGF-1 (1914 ng/mL, reference range by age for IGF-I levels is 83 ~ 221 ng/mL) and GH (80.3 ng/mL) levels. GH was not suppressed with 75 g glucose loading, (after 60 min: 131 ng/mL; after 120 min: 46.9 ng/mL).

### Imaging studies

**Fig. 2:**
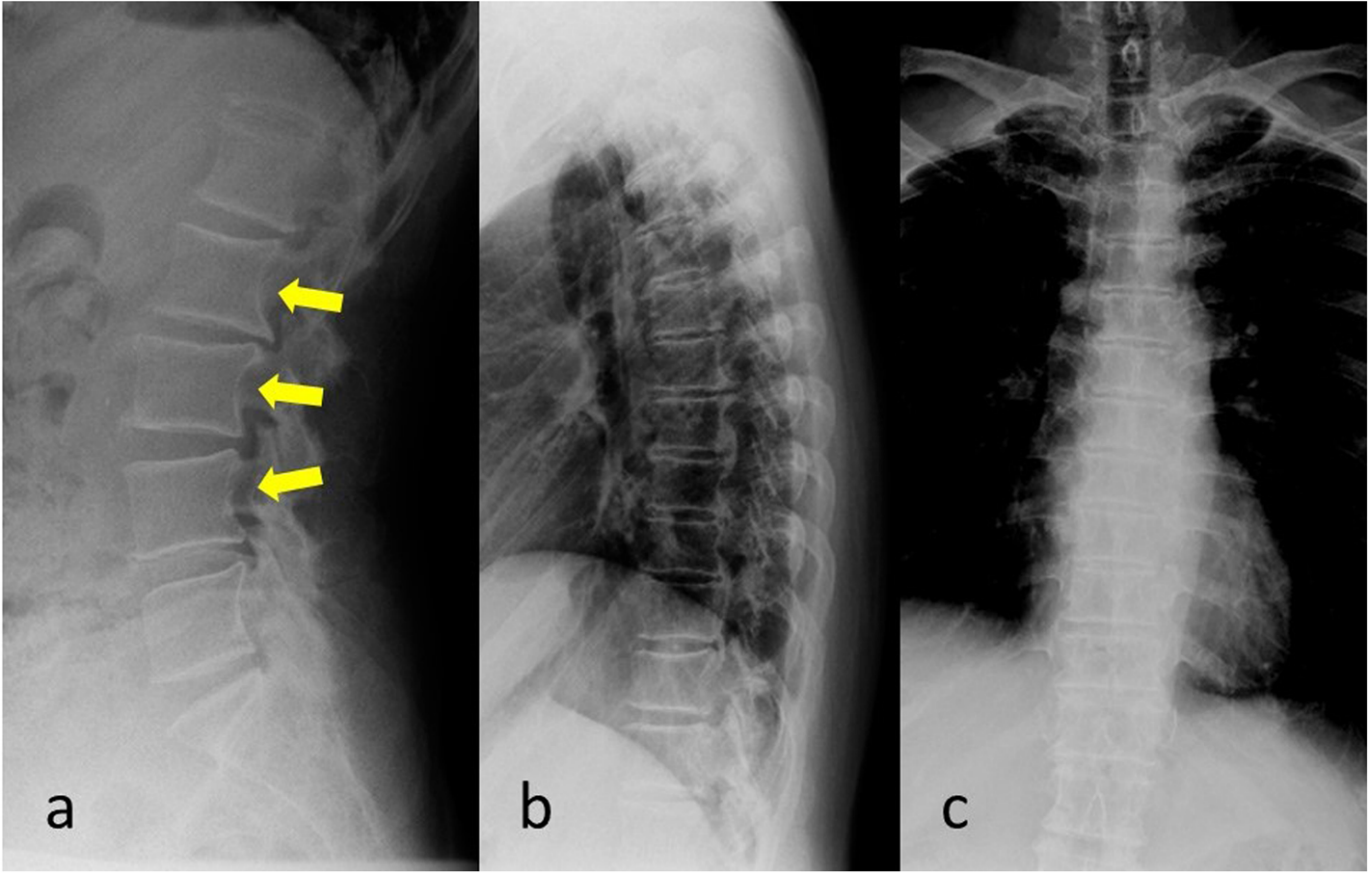
**a**-**c**: Preoperative lumber and thoracic X-ray images. Scalloping in some vertebrae was observed, as indicated by arrowheads on a lateral view of the lumbar X-ray. On thoracic spine X-ray there were no findings of DISH such as more than three successive bone bridges, and there were no anterior or posterior osteophytes with a biconcave appearance such as Erdheim spondylosis in the thoracic spine

X-ray images showed characteristic findings of acromegaly. Scalloping in some vertebrae was observed, as indicated by arrowheads on a lateral view of the lumbar X-ray in Fig. 2a [10]. On thoracic spine X-ray there were no findings of diffuse idiopathic skeletal hyperostosis (DISH) such as more than three successive bone bridges, and there were no anterior or posterior osteophytes with a biconcave appearance such as Erdheim spondylosis in the thoracic spine (Fig. 2b, c).

**Fig. 3:**
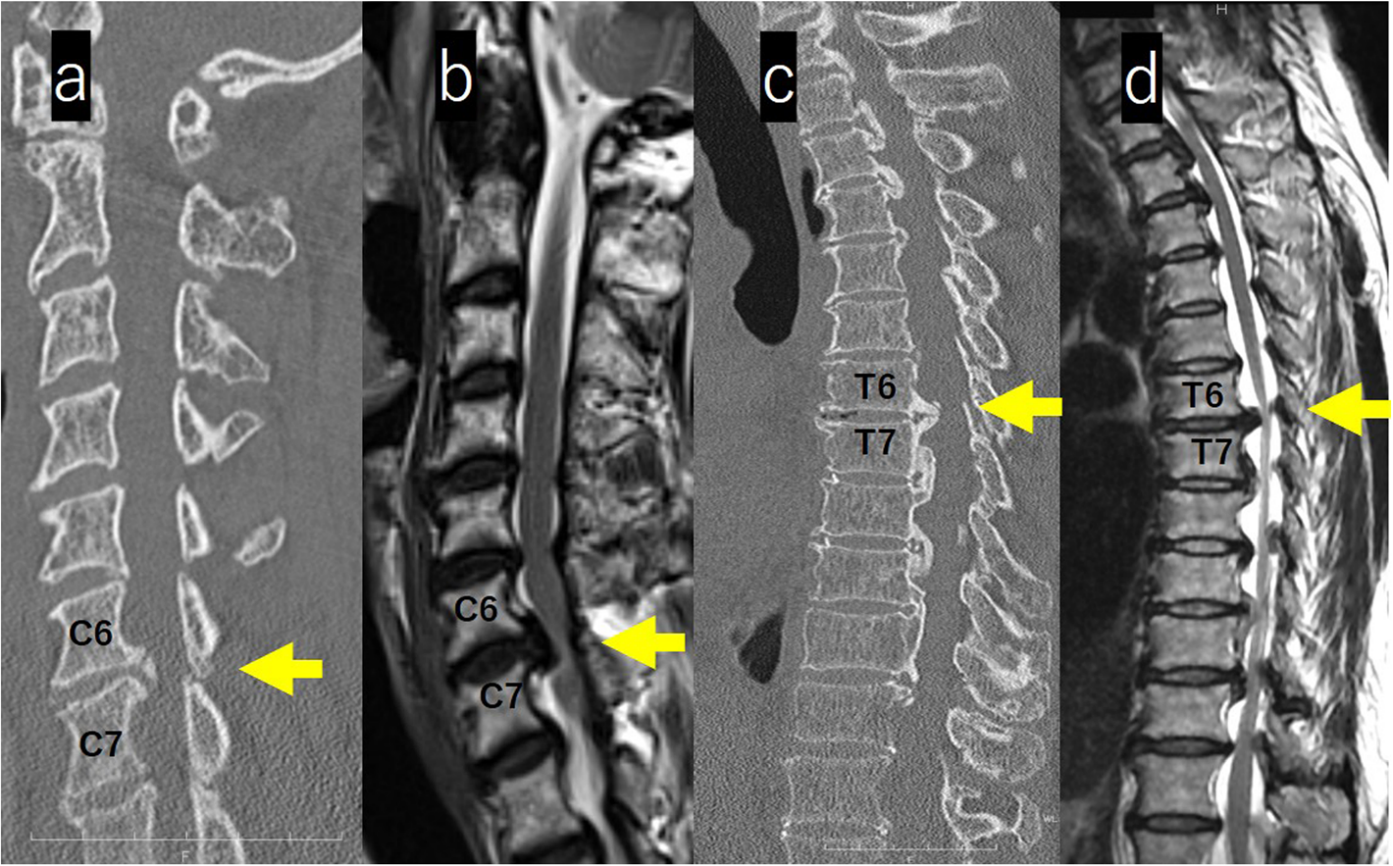
**a**-**d**: MRI and CT images of the cervical and thoracic spine. CT images of the whole spine, isolated OPLL was observed from the lower cervical vertebra to the middle thoracic vertebra. At T6/7, a Beak-shaped protrusion into the spinal canal was observed (**a**, **c**). MRI revealed stenosis with a spinal cade intensity change at C6/7 and extensive spinal cord compression with a spinal cade intensity change in the thoracic spine (**b**, **d**)

On computed tomography (CT) images of the whole spine, isolated OPLL was observed from the lower cervical vertebra to the middle thoracic vertebra. At T6/7, a beak-shaped protrusion into the spinal canal was observed (Figs. 3a, c).

**Fig. 4:**
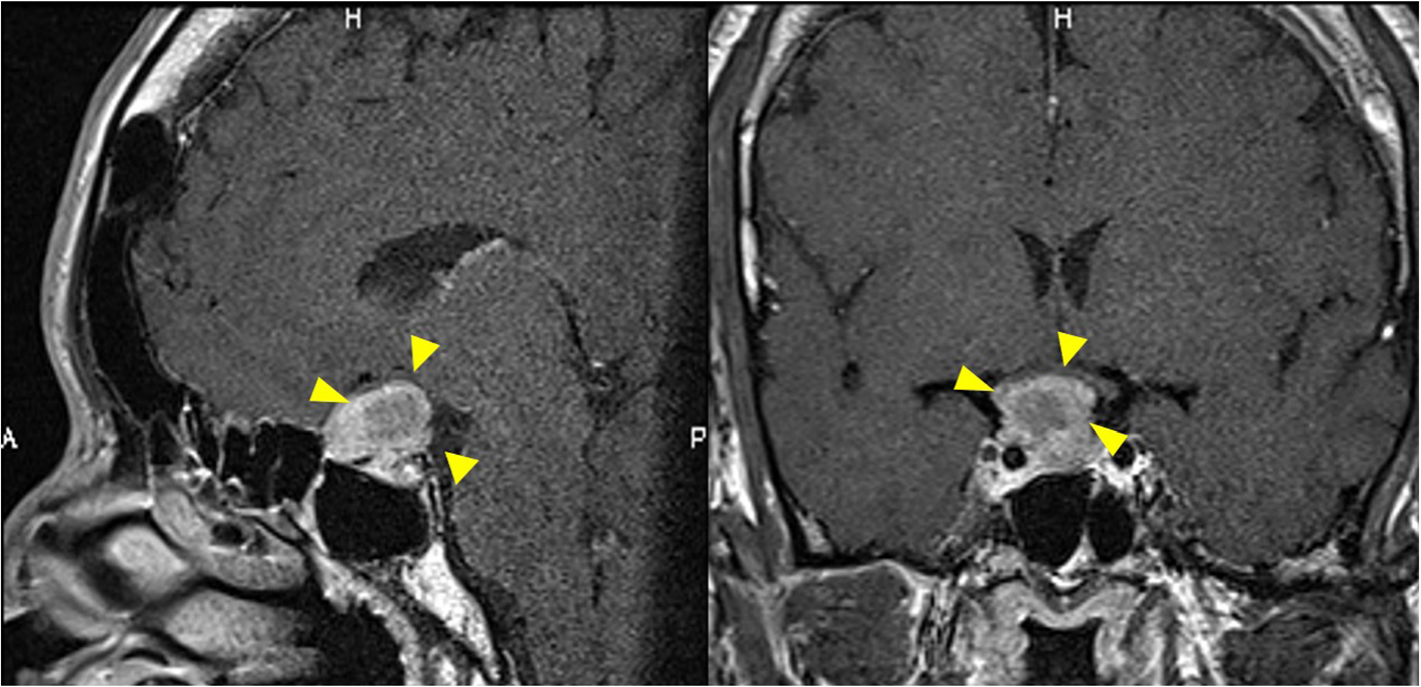
Preoperative contrast-enhanced MRI of the brain. It showed mass without contrast effect on the right side of the anterior pituitary gland (size, 10 mm × 12 mm × 10 mm) with suprasellar and parasellar extension

Magnetic resonance imaging (MRI) of the whole spine revealed stenosis with an intensity change in spinal cord at C6/7 and extensive spinal cord compression in the thoracic spine (Figs. 3b, d). Contrast-enhanced MRI of the brain showed a mass without contrast enhancement on the right side of the anterior pituitary gland (size, 10 mm × 12 mm × 10 mm) with suprasellar and parasailer extension; the image revealed a pituitary macroadenoma (Fig. 4).

### Posthospitalization course

The patient’s most remarkable symptom was paraplegia, and we diagnosed myelopathy due to thoracic OPLL. Furthermore, we suspected acromegaly because of the characteristic facial features and blood test findings (ALP 432 IU/L, GH 80.3 ng/ml, IGF-1914 ng/ml). Contrast-enhanced MRI of the brain performed by the endocrinology department showed a pituitary adenoma (Fig. 4). The patient’s symptoms satisfied the diagnostic criteria for acromegaly. Neurosurgery was scheduled for the pituitary adenoma, but spinal surgery was prioritized due to fear of exacerbation of neurological symptoms.

**Fig. 5:**
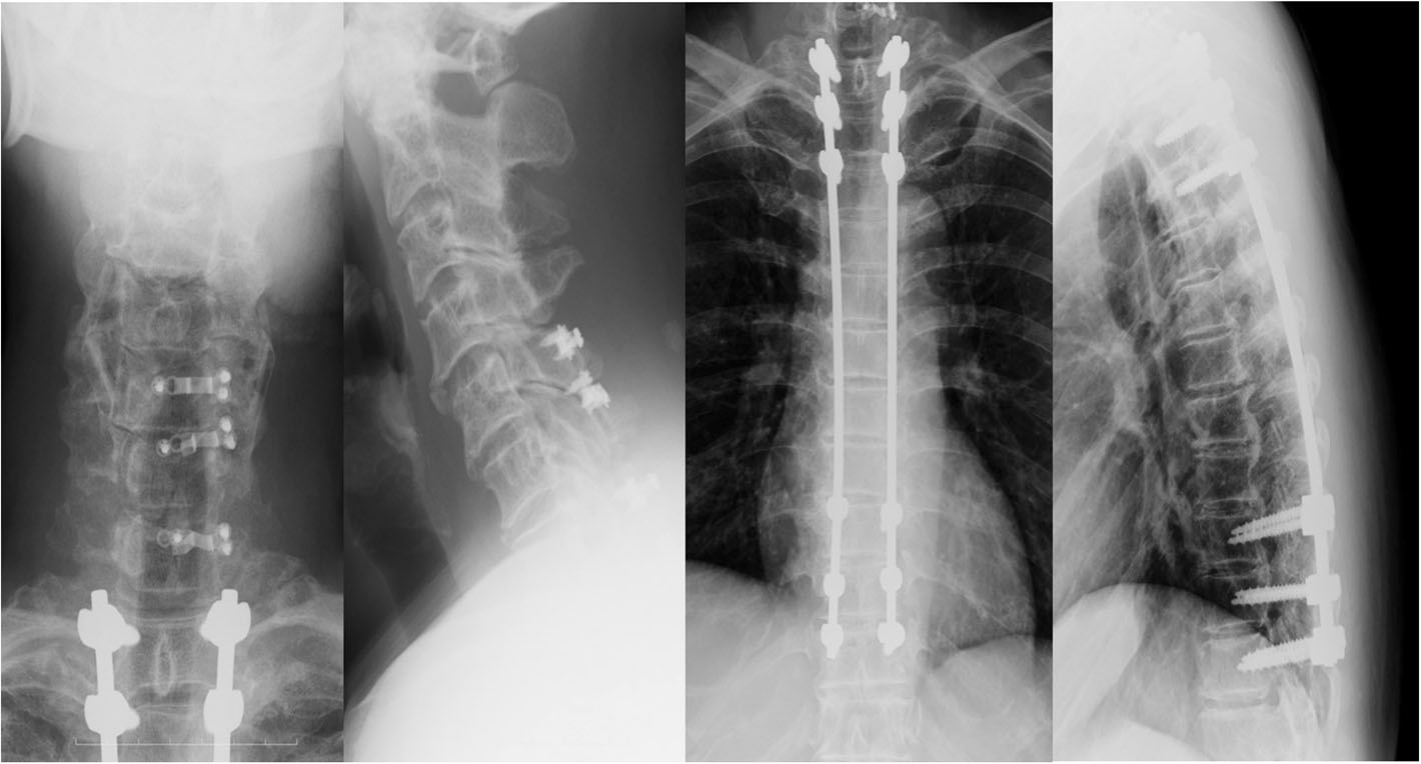
Postoperative cervical and thoracic lateral X-ray images. T1–3 and T6–11 Laminectomy and T1–11 posterior fixation were performed. And also, C5–7 open-door laminoplasty was performed with lamina plate

The neurological findings suggested spinal cord lesions below the T10 level, and T6/7 was judged to be the location of the primary lesion. T1–3 and T6–11 laminectomy and T1–11 posterior fixation were performed. Additionally, there was severe spinal cord compression in C6/7, and open-door laminoplasty with a lamina plate was performed for C5–7 (Fig. 5).

### Postoperative course

**Fig. 6:**
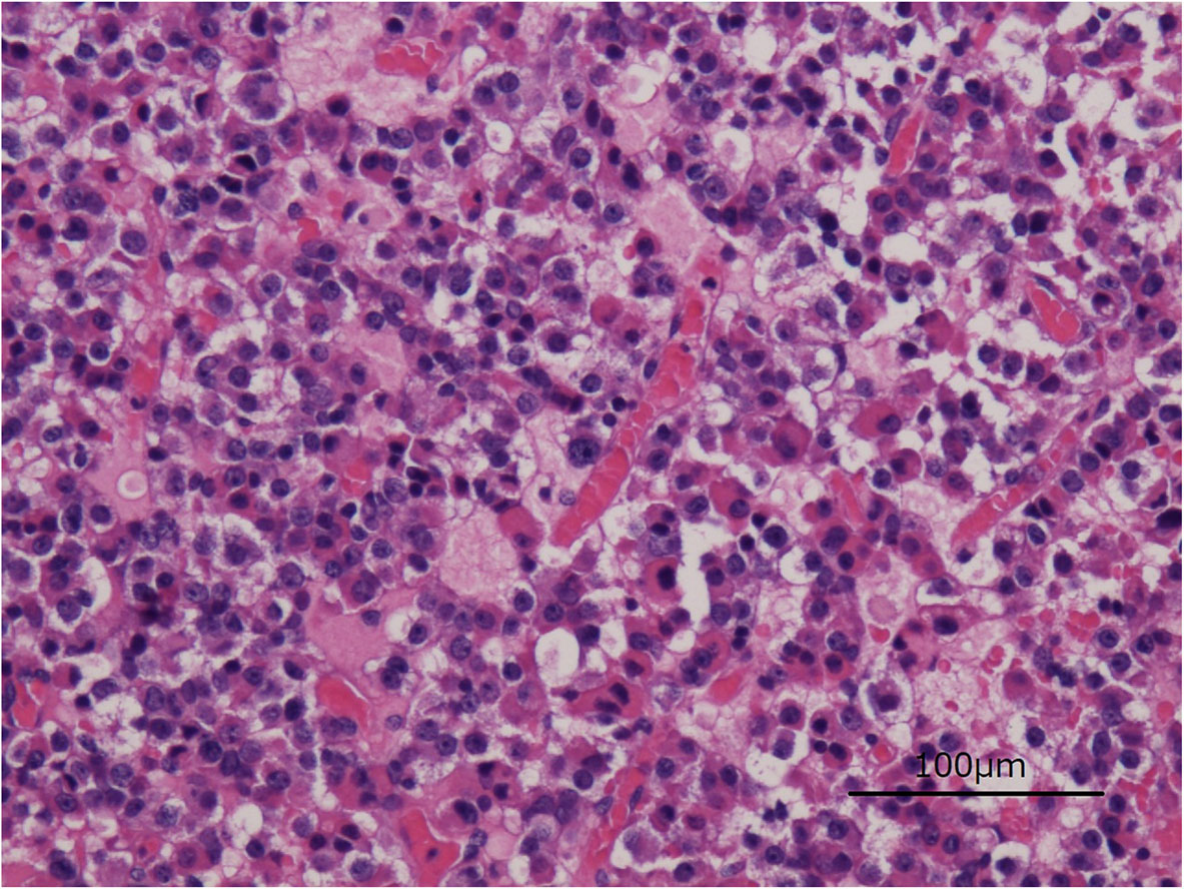
Pathological image of a pituitary adenoma (H&E, × 400). A sheet of monotonous cells with round nuclei and loss of normal lobular patterns of the pituitary adenoma. Using a microscope (BX51, Olympus, Tokyo, Japan) and an objective lens of × 20, the figure was taken under the settings of 1280 × 960 pixel (Camera, DS-F1, Nikon, Tokyo, Japan; Detector, Digital Sight DS-L2, Nikon, Tokyo, Japan)

On the third day after the operation, rehabilitation was started with a cervical collar and hard thoracolumbar corset. The postoperative course was good, the paralysis gradually improved, and the patient was able to walk alone indoors. She was discharged 3 weeks after the operation. There have been no adverse or unanticipated events. Two months after the spinal surgery, resection of the pituitary adenoma was performed in the neurosurgery department, and the patient is still under observation. A pathological image (H&E, × 400) is provided and showed a sheet of monotonous cells with round nuclei and loss of normal lobular patterns of the pituitary adenoma (Fig. 6). Staining for TSH and ACTH was negative. Staining for PRL was positive in 10% of the resected pituitary tissue. Staining for CAM5.2 was diffusely positive in many cells and several cells had fibrous bodies. These findings are consistent with growth hormone-secreting pituitary adenomas. We did not have other antibodies, so it was difficult to classify the pituitary adenoma in detail.

## Discussion and conclusions

Spinal and peripheral joint abnormalities are caused by GH hypersecretion in patients with acromegaly due to the secretion of IGF-1 from the liver by the actions of GH, which activates osteoblasts and fibroblasts [11]. Furthermore, it has been pointed out that the response to GH may be involved in the onset of ossification of the spinal ligament [12]. For instance, it has been reported that 20% of patients with acromegaly have diffuse idiopathic skeletal hyperostosis [13]. The prevalence of DISH in patients with cervical OPLL is 48.7% [14], and it is suggested that acromegaly and OPLL are strongly related. Furthermore, it has also been reported that serum growth hormone-binding protein (GHBP) levels were significantly higher in an OPLL group than in age-matched controls, whereas there was no significant difference between the two groups in serum levels of GH, IGF-1, or insulin-like growth factor 2 (IGF-2). These results, taken together with the hypothesis that serum levels of GHBP reflects the number of GH receptors in tissue, suggest that GH receptors are increased in patients with OPLL [6]. Moreover, in cultured ligament cells of OPLL patients and non-OPLL patients, IGF-I has the effect of inducing more ossification differentiation on OPLL patient ligament cells, and IGF-I is considered to be involved as a local factor of ossification in OPLL patients [7]. All these reports suggest a relationship between acromegaly and OPLL.

However, the annual incidence rates of acromegaly range between 2 and 11 per 1 million [8, 9], so we rarely encountered this disease. To the best of our knowledge, only three cases of OPLL and four cases of ossification of the ligament of the flavum (OLF) have been reported in a Japanese article and a paper published in PubMed, and there is only one case report of OLF [15]. All reported cases were of untreated or poorly controlled disease despite treatment and reported a long duration until a diagnosis was obtained. Even in our case, the facial features characteristic of acromegaly had appeared at least 9 years ago, as seen in past photographs of the patient (Figs.1a-c). This suggested exposure to excessive GH and IGF-1 for an extended period. It is possible that early diagnosis and treatment of acromegaly may suppress the progression of the ossification of ligaments, which is a subject for future study.

In addition, acromegaly may cause serious complications such as cardiovascular disorders, cerebrovascular disorders, malignant tumors, and sleep apnea, resulting in a poor prognosis [16–19]. The standardized mortality ratio of these patients is 1.2 to 3.6 compared to that of normal subjects [19–21]. However, the prognosis is significantly improved by early diagnosis and appropriate treatment [19, 22, 23]. Early detection is critical not only for the suppression of ossification but also from the perspective of improving prognosis. Symptoms that trigger the diagnosis are often facial changes and hypertrophy of the extremities [24]. Therefore, orthopedic surgeons and neurosurgeons need to keep in mind the characteristics of acromegaly.

We reported a case of acromegaly that was detected after the diagnosis of OPLL. Early diagnosis and treatment of acromegaly improve life prognosis and reduce exposure to GH and IGF-1 through early intervention and seem to suppress the progression of ligament ossification and avoid severe myelopathy. Orthopedic surgeons and neurosurgeons need to keep in mind that acromegaly is associated with bone/joint lesions and the ossification of the spinal ligament and should aim to diagnose acromegaly early.

## Data Availability

The datasets are available from the corresponding author on reasonable request.

## Abbreviations

GH: Growth hormone
IGF-1: Insulin-like growth factor 1
OPLL: Ossification of the posterior longitudinal ligament
OGTT: Oral glucose tolerance test
ALP: Alkaline Phosphatase
DISH: Diffuse Idiopathic Skeletal Hyperostosis
CT: Computed tomography
MRI: Magnetic resonance imaging
GHBP: Growth hormone-binding protein
IGF-2: Insulin-like growth factor 2
OLF: Ossification of the ligament of the flavum
